# Complete genome sequence of *Cellulomonas* sp., strain ES6, a chromate-reducing bacterium isolated from chromium-contaminated subsurface sediment

**DOI:** 10.1128/MRA.00495-23

**Published:** 2023-09-08

**Authors:** Katheryn R. Perea, Linda C. DeVeaux, Brady D. Lee, Nathaniel A. Losey

**Affiliations:** 1 Biology Department, New Mexico Institute of Mining and Technology, Socorro, New Mexico, USA; 2 Earth, Biological and Quantitative Systems Science Division, Savannah River National Laboratory, Aiken, South Carolina, USA; Wellesley College, Wellesley, Massachusetts, USA

**Keywords:** genomes, environmental microbiology

## Abstract

*Cellulomonas* sp. strain ES6 is a chromate-reducing bacterium isolated from chromium contaminated subsurface sediment. Illumina MiSeq and Oxford Nanopore sequencing were used to assemble the genome sequence which consisted of a single circular chromosome of 4.13 Mb, contained 3,960 protein encoding genes and with an overall G + C content 75.38%.

## ANNOUNCEMENT

*Cellulomonas* sp. strain ES6 was isolated from a mixed enrichment sampled from a sediment core at the edge of a dichromate plume (1). The sediment core was 1 ft long, 4 in. diameter, and collected at a depth of 47.5 ft from well 199-H5-15 (longitude 119.48452 and latitude 46.69895) at the Department of Energy’s Hanford Site (2). Initial enrichment of chromium-reducing microbes was performed using simulated groundwater media, and isolate ES6 was recovered by serial dilution and streak plating on tryptic soy agar (TSA, Difco Laboratories) (1). Further studies of strain ES6 found useful traits including chromate reduction, uranium precipitation via phosphate release, and ferric iron reduction (3–5). The ability of strain ES6 to alter the mobility of contaminants may be useful for biological remediation (6). A partial 478 bp fragment of the 16S rRNA gene of strain ES6 (GenBank #AY617100) was previously sequenced by others using unspecified primers and shares 99.56% sequence identity to the 16S rRNA gene of *Cellulomonas hominis* (GenBank #X82598.1) (1).

Strain ES6 was aerobically grown on TSA plates at room temperature for 2–3 days. DNA extraction was performed using MoBio Powersoil DNA Extraction Kit (Qiagen) according to manufacturer’s instructions except 100 mg wet cell mass was used instead of soil, and then extracted DNA was used for both Illumina and Nanopore sequencing. Nextera XT DNA Library Preparation Kit (Illumina) was used for library preparation followed by sequencing of genomic DNA by paired-end sequencing (2 × 250 bp) on an Illumina MiSeq instrument at Argonne National Laboratory Environmental Sample Preparation and Sequencing Facility. A total of 14,777,074 pairs of reads were acquired. The paired reads were processed using Trim Galore v0.6.10 (https://github.com/FelixKrueger/TrimGalore) to remove adapters and pairs of sequences with lengths under 160 bps or with quality scores <30. After quality control processing, 10,363,395 pairs of reads (mean length = 250 bp) were used for assembly.

Approximately, 360 ng of DNA was prepared using the Rapid Barcode Sequencing Kit (SQK-RBK004, Oxford Nanopore Technologies, Oxford, UK) and sequenced on R9.4.1 flow cell using a MinION MkB. A total of 77,182 sequences were acquired and processed using Filtlong (https://github.com/rrwick/Filtlong) with default settings. After processing, a total of 21,670 sequences were retained with sequence lengths from 5,380 to 169,051 bp (mean = 23,073 bp). Evaluation of both long and short reads was performed using FastQC v0.12.0 (https://www.bioinformatics.babraham.ac.uk/projects/fastqc/).

Genome assembly, overlap removal, and sequence rotation were performed using Unicycler (v0.5) run in hybrid assembly mode with default settings (7). The resulting assembly was a single circular contig of 4,124,887 bp with total GC content of 75.38%. The quality of the final assembly was evaluated using CheckM (v1.0.18) (8). Genome annotation of the final assembly was performed using PGAP (v6.5) (9).

CheckM results for the assembly were 97.11% for completeness and 0.0% for contamination. Sequence coverage of Illumina MiSeq reads was approximately 1256.2×, while Oxford Nanopore reads coverage was 120.2×. The PGAP annotation produced gene annotations of 3,790 genes, 3,640 putative protein coding genes, 45 tRNAs, 6 rRNA genes, 3 ncRNAs, and 96 pseudogenes.

## Data Availability

This Whole Genome Shotgun project is available from GenBank under the accession no. CP125655. The version described in this paper is the first version CP125655.1. The associated BioProject accession number is PRJNA952319, the associated BioSample accession number is SAMN34072617, the accession number for the associated Illumina MiSeq SRA is SRX20326346, and the accession number for the Nanopore SRA is SRX20326345.
